# Cognition and dementia in older patients with epilepsy

**DOI:** 10.1093/brain/awy022

**Published:** 2018-02-28

**Authors:** Arjune Sen, Valentina Capelli, Masud Husain

**Affiliations:** 1 Oxford Epilepsy Research Group, NIHR Biomedical Research Centre, Nuffield Department Clinical Neurosciences, John Radcliffe Hospital, Oxford, UK; 2 Department of Experimental Psychology, University of Oxford, UK

**Keywords:** neuropsychology, cognitive function, brain imaging, electrophysiology

## Abstract

With advances in healthcare and an ageing population, the number of older adults with epilepsy is set to rise substantially across the world. In developed countries the highest incidence of epilepsy is already in people over 65 and, as life expectancy increases, individuals who developed epilepsy at a young age are also living longer. Recent findings show that older persons with epilepsy are more likely to suffer from cognitive dysfunction and that there might be an important bidirectional relationship between epilepsy and dementia. Thus some people with epilepsy may be at a higher risk of developing dementia, while individuals with some forms of dementia, particularly Alzheimer’s disease and vascular dementia, are at significantly higher risk of developing epilepsy. Consistent with this emerging view, epidemiological findings reveal that people with epilepsy and individuals with Alzheimer’s disease share common risk factors. Recent studies in Alzheimer’s disease and late-onset epilepsy also suggest common pathological links mediated by underlying vascular changes and/or tau pathology. Meanwhile electrophysiological and neuroimaging investigations in epilepsy, Alzheimer’s disease, and vascular dementia have focused interest on network level dysfunction, which might be important in mediating cognitive dysfunction across all three of these conditions. In this review we consider whether seizures promote dementia, whether dementia causes seizures, or if common underlying pathophysiological mechanisms cause both. We examine the evidence that cognitive impairment is associated with epilepsy in older people (aged over 65) and the prognosis for patients with epilepsy developing dementia, with a specific emphasis on common mechanisms that might underlie the cognitive deficits observed in epilepsy and Alzheimer’s disease. Our analyses suggest that there is considerable intersection between epilepsy, Alzheimer’s disease and cerebrovascular disease raising the possibility that better understanding of shared mechanisms in these conditions might help to ameliorate not just seizures, but also epileptogenesis and cognitive dysfunction.

## Teaser

Older people with epilepsy are more likely to suffer from dementia, while individuals with dementia are at higher risk of developing epilepsy. Sen *et al.* consider common mechanisms that might underlie the cognitive deficits observed in both groups by examining findings from epidemiological, neuropsychological, molecular, electrophysiological and brain imaging perspectives.

## Introduction

Around 65 million people worldwide have epilepsy, with ∼80% living in developing regions (Birbeck, 2010; Ngugi *et al.*, 2010). In the UK >600 000 people, i.e. almost 1 in 100 (Joint Epilepsy Council, 2011) and in the USA >3 million people or 0.84 in 100 (Helmers *et al.*, 2015) have the disorder. Several studies have consistently shown that the peak incidence is higher in the older population, rising from the age of 65 (Annegers *et al.*, 1999; Hussain *et al.*, 2006). In fact ∼25% of new-onset epilepsies are diagnosed after this age (Joint Epilepsy Council, 2011). Given that the global population aged >65 will increase by ∼400 million to reach almost 1 billion by 2030, the number of older adults with epilepsy is expected to rise substantially.

The population of older adults with epilepsy (defined here as >65 years) consists of two main groups: those who have had epilepsy for many years and, owing to improvements in healthcare, are now living to older age, and those who develop epilepsy *de novo* in later life. While several underlying causes may contribute to new-onset epilepsy in the elderly (Stefan, 2011) cerebrovascular disease accounts for 50–70% of cases and is the single most common cause (Brodie *et al.*, 2009; Choi *et al.*, 2017). Recent work from the USA has reported that the incident risk for epilepsy is highest in people with cerebrovascular disease aged 75–79, with African Americans at particularly high risk (Choi *et al.*, 2017). The incidence of epilepsy was highest of all in older patients who had experienced a stroke. Others have shown that in the first year after a stroke the risk of developing epilepsy can increase 20-fold (Brodie *et al.*, 2009). The exact pathophysiology of stroke-related epilepsy is not established, but intracerebral haemorrhage, haemorrhagic transformation of ischaemic stroke, greater stroke severity, cortical involvement and venous sinus thrombosis all increase the risk of seizures (Conrad *et al.*, 2013; Zhang *et al.*, 2014).

Older people who experience head trauma are also around 2.5 times more likely to develop post-traumatic epilepsy than their younger counterparts and up to 20% of epilepsy in the elderly may be attributable to head injury (Annegers *et al.*, 1998; Bruns and Hauser, 2003). Dementia and neurodegenerative disorders account for a further 10–20% of late-onset cases of epilepsy (Stefan, 2011), with much of the research to date focusing on Alzheimer’s disease. Patients with Alzheimer’s disease aged ≥65 years have up to a 10-fold higher risk of epilepsy (Hommet *et al.*, 2008; Pandis and Scarmeas, 2012). Other causes of dementia have received far less attention, but one large study in the UK reported that in people over 65, individuals classified as having vascular dementia had a similar likelihood of developing seizures or epilepsy as those with Alzheimer’s disease (Imfeld *et al.*, 2013).

Importantly, some findings also suggest that older patients with epilepsy are at a higher risk of developing cognitive impairment and ultimately dementia (van Duijn *et al.*, 1991; Breteler *et al.*, 1995). Why should that be the case? In this review we consider whether seizures promote dementia, whether dementia causes seizures, or if common underlying pathophysiological mechanisms are responsible for both. First, we consider the evidence regarding cognitive function and the potential higher risk of dementia in older patients (aged >65 years) with epilepsy. We then turn to findings that suggest there might be a bidirectional link between epilepsy and dementia such that patients with dementia also seem to be at greater risk of developing seizures (Subota *et al.*, 2017). We discuss the evidence for common lifestyle and vascular risk factors in epilepsy and dementia, and then consider potential shared molecular links, including new evidence for tau pathology in older patients with epilepsy (Sen *et al.*, 2007*a*; Thom *et al.*, 2011; Tai *et al.*, 2016). Finally, we examine emerging evidence which suggests that widespread, brain network changes in Alzheimer’s disease (with our without concomitant vascular pathology) and epilepsy might contribute to cognitive dysfunction in these conditions (Wandschneider *et al.*, 2014; Canter *et al.*, 2016; Palop and Mucke, 2016; Chong *et al.*, 2017), including the remote effects of interictal epileptiform discharges (IEDs) (Kleen *et al.*, 2013; Gelinas *et al.*, 2016; Ung *et al.*, 2017).

Our review of these disparate findings leads to the conclusion that although current data do not allow us to make definitive mechanistic inferences, they do show that this is an important area for investigation that has potential application to both patients with epilepsy and dementia. It is now well established that many patients who are diagnosed clinically to have Alzheimer’s disease have mixed pathology with concurrent cerebrovascular changes at post-mortem, and vice versa (Rahimi and Kovacs, 2014)*.* Thus, targeting lifestyle and vascular risk factors might offer important therapeutic opportunities to modify the disease processes underlying these conditions as well as the progression of cognitive decline in people with epilepsy

## Cognitive function in older patients with epilepsy

### Studies of cognitive impairment in older patients with epilepsy

There are surprisingly few systematic studies that have addressed the issue of cognitive function specifically in the elderly epilepsy population (Martin *et al.*, 2005; Griffith *et al.*, 2006, 2007; Piazzini *et al.*, 2006; Witt *et al.*, 2014; Miller *et al.*, 2016). Most of these are cross-sectional investigations that tested small samples of patients with young-onset epilepsy (Table 1), with only one study that has reported on late-onset epilepsy (Witt *et al.*, 2014). Nevertheless, the work that has been published indicates that overall older adults with epilepsy have greater deficits compared to healthy older people across cognitive domains, but especially in short and long-term visual and verbal memory, executive functions, attention and psychomotor, or processing speed.

**Table 1:** Results of studies of cognitive impairment in older patients with epilepsy

Study and sample	Cognitive domains	Neuropsychological tests	Findings
Martin *et al.*, 2005 *n* = 25 cases (mean age 64.6, SD 3.9) versus 27 healthy controls	Global cognitive functioning	Mattis DRS	Patients with epilepsy impaired on all tests Patients on AED polytherapy more impaired than those on monotherapy
	Immediate and long-term memory	*Logical Memory*: Immediate and Delayed recall subtests from the WMS-III	
	Verbal fluency	COWAT	

Griffith *et al.*, 2006 *n* = 26 (mean age 64.7, SD 3.8) versus 26 healthy controls and 26 MCI patients	Global cognitive functioning	Mattis DRS	Patients with epilepsy impaired with respect to healthy older controls. Short-memory impairment similar in epilepsy and MCI Patients with epilepsy on AED polytherapy more impaired than patients with MCI
	Immediate and long-term memory	*Logical Memory*: Immediate and Delayed recall subtests from the WMS-III	
	Lexical ability	CFL word fluency test	

Griffith *et al.*, 2007 *n* = 17 (mean age 67.7, SD 4.2) versus 17 healthy controls	Global cognitive functioning	Mattis DRS	General stability in neurocognitive performance for patients with epilepsy after 3 years, although their performance continued to be below that of matched healthy adults
	Immediate and long-term memory	*Logical Memory:* Immediate and Delayed recall subtests from the WMS-III	
	Lexical ability	COWAT	
	Executive control.	Executive Interview Test (EXIT-25)	

Piazzini *et al.*, 2006 *n* = 40 (mean age 67.0, SD 5.9) versus 40 healthy controls	Intelligence	Raven’s Colored Progressive Matrices	Patients with epilepsy impaired on all tests Patients on AED polytherapy more impaired than those on monotherapy
	Selected and divided attention	Trail Making Test	
	Abstraction	Attentional Matrices	
	Short and long-term verbal and visual memory	Story Test Rey–Osterrieth Complex Figure	
	Learning	Digit Span	
	Language	Verbal Fluency Test	
	Aphasia	Token Test	

Witt *et al.*, 2014 *n* = 257 (mean age 71.5, SD 7.2)	Executive function	EpiTrack	Objective impairment in executive functions before initiation of AEDs treatment
	Quality of life	Quality of Life in Epilepsy (QOLIE)-31	
	Subjective ratings of cognition	Portland Neurotoxicity Scale (PNS)	

Miller *et al.*, 2016 *n* = 38 (mean age 65.2, SD 7.9) versus 29 healthy controls	Global cognition	Mattis DRS MMSE	Patients with epilepsy impaired on most domains (visuospatial skills relatively intact) Anxiety or AED polytherapy was associated with greater impairment on some cognitive domains
	Verbal memory	Hopkins verbal learning test – Revised Verbal paired associates	
	Visual memory	Brief visuospatial memory test – Revised Trail making test – A	
	Attention / psychomotor speed	Digit symbol coding	
	Executive function	Trail making test – B Controlled word association Rey Complex Figure (organization score)	
	Language	Boston naming test Animal fluency	
	Visuospatial	Rey Complex Figure Judgment of line orientation	

COWAT = Controlled Oral Word Association Test; DRS = dementia rating scale; MCI = mild cognitive impairment; MMSE = Mini-Mental State Examination; WMS-III = Wechsler Memory Scale.

One study has documented the severity of cognitive deficits in a large group (*n* = 257) of older people [mean age: 71.5, standard deviation (SD) 7.2] with new-onset focal epilepsy who were assessed before initiation of anti-epileptic drug (AED) treatment (Witt *et al.*, 2014). Approximately one-third (*n* = 88) had suffered a cerebral infarct and another third (*n* = 99) had cerebrovascular disease. Over 80% (*n* = 209) suffered from focal seizures with impairment of awareness and just over 50% (*n* = 139) suffered generalized tonic-clonic seizures. Cognitive function was tested using a screening tool specifically designed to detect and monitor executive function in patients with epilepsy (EpiTrack). This includes tests of response inhibition, visuomotor speed, mental flexibility, visuomotor planning, verbal fluency and working memory (Lutz and Helmstaedter, 2005). Performance was compared to age-corrected norms from 689 healthy control subjects. In addition, participants also completed the Portland Neurotoxicity Scale (PNS), a patient-based survey of cognitive and somatomotor symptoms and the Quality of Life in Epilepsy (QOLIE)-31 questionnaire.

The results revealed that many older individuals with new-onset focal epilepsy were cognitively impaired before initiation of AEDs, with 43% markedly affected, 35% were unimpaired while 6% scored above average. Greater deficits were associated with cerebral infarction or cerebrovascular aetiology, neurological comorbidity and higher body mass index. Subjective performance ratings indicated limited insight into cognitive impairments. These findings underline the importance of early cognitive screening to obtain a baseline assessment allowing quantification of any decline and to evaluate effects of subsequent pharmacological treatment. Overall, the limited existing studies on this topic show that older individuals with epilepsy appear to have significant deficits in cognitive function across the board.

### Accelerated long-term forgetting

In addition to systematic studies of cognitive function, there is also evidence of accelerated long-term forgetting in one group of patients with epilepsy, namely individuals with temporal lobe epilepsy (TLE). In this condition, memories appear to be normally encoded but are retained for only short intervals of time: weeks, days or even less (Martin *et al.*, 1991; Elliott *et al.*, 2014). Accelerated long-term forgetting, specifically in older patients with epilepsy, has not been characterized in great detail and is difficult to interepret in the context of the controversial literature on the effects of normal ageing on forgetting (Elliott *et al.*, 2014). However, one group of patients in which accelerated long-term forgetting has been reported in an older age group (mean age of onset = 57 years) is those with transient epileptic amnesia (Manes *et al.*, 2005; Butler *et al.*, 2007; Butler and Zeman, 2008). In transient epileptic amnesia, amnestic episodes usually last less than 1 h, may be accompanied by olfactory hallucinations and automatisms and brief loss of responsiveness, and the EEG often indicates medial temporal lobe dysfunction (Butler *et al.*, 2007).

Patients experience a panoply of symptoms that often occur on awakening such as brief recurrent episodes of mixed anterograde and/or retrograde amnesia; difficulty in forming new memories; and in remembering events that occurred in the hours prior to the onset of the attack. Other cognitive functions have been reported as unaffected during typical episodes (Butler and Zeman, 2008). Scores on tests of general intelligence, language, executive function and visuospatial perception are also usually not significantly different from healthy people (Butler *et al.*, 2007). Patients with transient epileptic amnesia can also experience topographical amnesia: loss of spatial memory with difficulty recalling routes or places. In addition, they typically report a remote memory impairment, in which there is loss of memories for relevant autobiographical events in the more distant past (Butler and Zeman, 2008). Whether accelerated long-term forgetting and/or remote memory impairment might predispose to development of dementia remains to be definitively determined, but data on progression of cognitive deficits are perhaps strongest for those with TLE, as discussed in the next section.

### Progression of cognitive deficits in younger-onset epilepsy patients

Several investigations have now also reported that there is progression of cognitive deficits over short periods of time (3–4 years), in younger adults (<65 years old) with epilepsy (reviewed in Dodrill, 2004; Seidenberg *et al.*, 2007). The most extensively studied group is patients with TLE in whom it is now apparent that there are widespread brain structural changes beyond the medial temporal lobes (Keller and Roberts, 2008; Bell *et al.*, 2011; Dabbs *et al.*, 2012; Caciagli *et al.*, 2017) and that progression of cognitive impairment over time may occur in a significant proportion (Hermann *et al.*, 2006). While cognitive reserve (Hermann *et al.*, 2006; Lin *et al.*, 2012)—indexed by baseline IQ, years of education or occupational complexity—may mitigate some of these effects, duration of epilepsy appears to be an important factor in determining progression of cognitive impairment (Hermann *et al.*, 2006; Seidenberg *et al.*, 2007).

**Figure 1:**
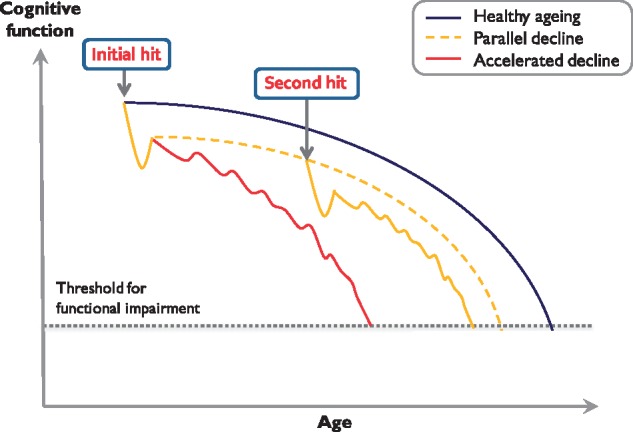
**Trajectories of cognitive decline with ageing.** The schematic illustrates how cognitive function in people with epilepsy might decline compared to healthy ageing (blue). One model proposes that an initial brain insult (‘initial hit’) leads to cognitive decline in epilepsy patients simply running parallel to but below the normal trajectory (dashed yellow). These individuals start from lower cognitive performance and also reach the threshold for functional impairment or dementia earlier. An alternative model is that while an initial hit might be a neurodevelopmental disorder or traumatic brain injury, subsequent development of epilepsy is in effect a ‘second hit’, which leads to further deviation from the normal trajectory (solid yellow). A third proposal is that, with increasing time, the trajectory of cognitive decline in people with epilepsy deviates further from that in healthy individuals leading to accelerated cognitive ageing (red). Inspired by a figure used by Breuer *et al.* (2016).

Whether the progression represents genuine accelerated ageing over time (Seidenberg *et al.*, 2007; Sung *et al.*, 2013) or simply the initial ‘hit’ of epilepsy having a negative neurodevelopmental impact (Helmstaedter and Elger, 2009) is a matter of considerable debate (Lin *et al.*, 2012; Breuer *et al.*, 2016). According to one view of accelerated ageing, the trajectory of cognitive decline with ageing in people with epilepsy deviates further from that in healthy individuals as time passes (Fig. 1). That is to say, there is continued cognitive decline over time or progression (Seidenberg *et al.*, 2007). This might be due to chronic accrual of underlying pathology (e.g. vascular) and/or the effects of epilepsy itself (ongoing overt seizures or interictal epileptiform discharges, IEDs). However, an alternative view is that an initial insult to the brain means that, in patients with epilepsy, cognitive decline simply runs below, but parallel to, the normal trajectory of cognitive change with ageing (Fig. 1). These individuals therefore start from a lower point and therefore reach thresholds for significant cognitive and functional impairment earlier than those without seizures (Helmstaedter and Elger, 2009). Others suggest there may be a ‘second hit’. According to this latter view some disruption to the brain (e.g. traumatic brain injury) or pre-existing brain abnormality represents the first hit on cognitive function, but subsequent development of epilepsy is effectively a ‘second hit’ that leads to further deviation from the normal trajectory of cognitive decline with ageing (Fig. 1) (Breuer *et al.*, 2016).

Evidence for progression of cognitive decline in epilepsy comes from a relatively small number of longitudinal studies in which the majority of patients had focal seizures, mostly TLE, with many being associated with evolution to bilateral tonic clonic seizures (Seidenberg *et al.*, 2007). In addition, most of these studies examined people who at baseline had a mean age of 31–37 years and were followed-up between 1 and 13 years. One investigation has reported on a small sample (*n* = 17) of older individuals who at baseline had a mean age of 64 years and were followed up 2–3 years later (Griffith *et al.*, 2007). In this study, participants were also assessed with the Executive Interview Test (EXIT-25) to assess executive function. Overall, cognitive deficits did not worsen in this group, although performance continued to be below that of matched healthy older adults. However, delayed logical memory showed a significantly deleterious trajectory. In addition, performance on the EXIT-25 also worsened, suggesting that older adults with epilepsy might experience greater decline in executive function over time.

A recent study by Breuer and co-workers screened 287 adult patients for cognitive deterioration compared to expected premorbid IQ (defined as ≥1 SD discrepancy between actual WAIS IQ and estimated premorbid IQ) (Breuer *et al.*, 2017). A group of 27 individuals fulfilled the criteria (mean age: 55.7; mean seizure duration: 21.8 years). More than 77% of them had associated comorbidities (including 52% cardiovascular, 14% cerebrovascular and 24% traumatic brain injury). Analyses revealed that the most prominent factors that accounted for the variance in cognitive deterioration were those that might impact on cognitive reserve: low premorbid IQ and education level, later age of seizure onset and older age (Breuer *et al.*, 2017). These findings would be consistent with a double hit model in which pre-existing low brain reserve makes the brain more vulnerable to a second hit from the development of epilepsy.

Hermann *et al.* (2007) sought to apply a taxonomic classification to the cognitive impairments in TLE by comparing 96 TLE patients with 82 healthy control participants. Using cluster analysis, they found that although all patients with TLE were impaired compared to controls, they could be further segregated. One group had minimal impairment (47%; although delayed memory, language, executive function and psychomotor processing speed were still significantly lower than controls). A second group had marked memory difficulties and evidence of relatively mild cognitive dysfunction across other domains (24%). Finally, a third group showed a global pattern of cognitive difficulties (29%; significantly poorer performance than controls and the first and second groups). Patients in this group had an earlier age of onset, were a little older (thereby indicating a longer duration of epilepsy) and were more likely to be on more AEDs than individuals in the other groups. A subgroup of participants was assessed again after 4 years. All had declined compared to controls, but the deterioration was more marked in Group 3 (Hermann *et al.*, 2007).

The initial work demonstrated that Group 3 had lower cerebral tissue and hippocampal volumes on MRI (Hermann *et al.*, 2007). A follow-up investigation showed that the cognitive subtypes could be differentiated through a wide variety of MRI measures suggesting that widespread neuroanatomical changes might underpin the differing patterns of cognitive deficit (Dabbs *et al.*, 2009). In both studies, the groups were aged 14–59 years. It remains to be established if there are neuroanatomical changes that might delineate differences between patients with, for example, late-onset TLE and memory difficulties compared to a cohort of individuals with Alzheimer’s disease. Nevertheless, it is clear that there is considerable heterogeneity within even one subgroup of patients with epilepsy. Thus generalizations about ‘single hit’, accelerated ageing and ‘double hit’ models might not be appropriate. Instead, the precise model that might explain cognitive trajectory might vary across individuals.

## Epilepsy and dementia in older patients: a bidirectional relationship?

Gowers first introduced the concept of epileptic dementia, implying that dementia and epilepsy might, in some subjects, be the consequence of the same underlying disorder (Gowers, 1881). The higher incidence of cognitive impairment in older people with epilepsy certainly raises questions as to whether these individuals might have increased rates of progression to dementia, particularly those with TLE (Höller and Trinka, 2014). As part of the EURODEM project, eight case-control studies that assessed the risks of developing Alzheimer’s disease in several medical conditions were reanalysed (van Duijn *et al.*, 1991). When compared with population-based controls, individuals with epilepsy had an increased relative risk of being diagnosed with Alzheimer’s disease at least 1 year after epilepsy diagnosis. The greatest risk for Alzheimer’s disease occurred in patients who had epilepsy for <10 years (relative risk 2.5) versus >10 years (relative risk 1.4). Importantly though, this increased risk appeared not to be related to the cumulative effect of longstanding seizures. A follow-up study based on three nationwide Dutch morbidity registers over the period 1980–89 investigated the risk of dementia for patients aged 50–75 years. Patients with epilepsy were found to have a relative risk of 1.5 of being diagnosed with dementia over an 8-year period (Breteler *et al.*, 1995).

The mechanisms underlying an increased likelihood of developing dementia have not been definitively established. Intriguingly, part of the risk might be attributable to the fact that patients with Alzheimer’s disease and/or vascular dementia are actually more likely to develop epilepsy (Hommet *et al.*, 2008; Imfeld *et al.*, 2013). In a UK study that examined the records of 22 084 patients (mean age ∼80 and half the sample with Alzheimer’s disease), after adjusting for baseline predictors of seizures, Alzheimer’s disease was associated with a significantly increased risk of seizures [hazard ratio (HR) 5.31, 95% confidence interval (CI) 3.97–7.10] (Cook *et al.*, 2015). In addition, patients with Alzheimer’s disease were at a higher risk of haemorrhagic stroke (HR 1.49, 95% CI 1.06–2.08), which is an independent risk factor for developing seizures. In Han Chinese patients with Alzheimer’s disease too, the risk of seizures is higher in Alzheimer’s disease than in age-matched controls (HR 1.85; 95% CI 1.40–2.90) (Cheng *et al.*, 2015).

Vascular dementia also increases the risk of seizures. Another large UK population-based study examined the records of 4438 cases with vascular dementia and 7086 patients with Alzheimer’s disease, comparing them to 11 524 dementia-free patients (mean age >80 in the three samples). This reported an odds ratio of developing seizures of 5.7 (95% CI 3.2–10.1) for vascular dementia and 6.6 (95% CI 4.1–10.6) for Alzheimer’s disease (Imfeld *et al.*, 2013). Thus, it might not simply be a case of epilepsy and associated risk factors, increasing the risk of dementia, but dementia—whether in the form of Alzheimer’s disease or vascular dementia—simultaneously increasing the risk of epilepsy (Helmstaedter and Witt, 2017).

These considerations raise important questions: do seizures cause dementia, does dementia cause seizures, or does something else cause both? Some recent work suggests that epilepsy could be considered a symptom rather than a disease in itself (Helmstaedter and Witt, 2017). It is argued that epilepsy is simply one manifestation of the underlying pathological process that might contribute to seizures, cognitive decline, psychological problems, systemic illness and, perhaps indirectly, psychosocial difficulties. Seizures can, for example, be observed in the prodromal phase of several neurodegenerative illnesses (Cretin *et al.*, 2017), and some studies in patients with mild cognitive impairment or Alzheimer’s disease have reported that cognitive decline may begin several years earlier in those individuals who suffer from seizures compared to those who do not (Amatniek *et al.*, 2006; Irizarry *et al.*, 2012; Vossel *et al.*, 2013). These findings have led some authors to consider whether there might be analogous processes occurring in patients with TLE who might progress to dementia (Höller and Trinka, 2014).

In patients with familial autosomal dominant early-onset Alzheimer’s disease (<50 years old) seizures are far more common than in typical late-onset Alzheimer’s disease, affecting >45% of cases, although rates vary with specific genetic mutations (Zarea *et al.*, 2016). This suggests that younger people with Alzheimer’s disease (aged 50–59) are at highest risk of developing seizures and therefore disease duration might not be crucial (Amatniek *et al.*, 2006). There is also some evidence that epilepsy in typical late-onset Alzheimer’s disease may be associated with faster progression of cognitive decline (Volicer *et al.*, 1995; Amatniek *et al.*, 2006), but large-scale longitudinal studies are required to confirm this. As highlighted in a comprehensive recent systematic review, much more work is needed to fully elucidate the epidemiology of epilepsy in dementia and also of dementia in epilepsy (Subota *et al.*, 2017). It remains unclear whether epilepsy and dementia simply share common risk factors, whether there is truly a bidirectional relationship between the two—or both. In the next section we examine evidence for common risk factors.

## Common risk factors for epilepsy and dementia

An important issue in dementia research is detection of disease at an early stage. The use of CSF (e.g. tau and amyloid-β levels) and neuroimaging biomarkers (e.g. hippocampal atrophy on structural MRI, temporoparietal hypometabolism on FDG PET, abnormal amyloid and tau PET imaging) to make the diagnosis of Alzheimer’s disease in the ‘preclinical’ state is one strategy that has gained widespread currency (Jack and Holtzman, 2013; Ahmed *et al.*, 2014). Similarly, the diagnosis of vascular dementia often rests on the presence of evidence of small vessel cerebrovascular change on MRI, while the clinical diagnosis of mixed Alzheimer’s disease/vascular dementia usually occurs when there are biomarkers suggesting the presence of both types of pathology (Harper *et al.*, 2014). Precisely how individuals with epilepsy with preclinical dementia might present or be detected remains to be established (Höller and Trinka, 2014). Indeed, it may well be that biomarkers for dementia in epilepsy might not be unique to people with epilepsy but rather reflect mixed pathologies, e.g. associated with Alzheimer’s disease and vascular dementia, as well as perhaps electrophysiological evidence of ongoing overt or covert abnormal electrical activity, e.g. IEDs.

**Figure 2:**
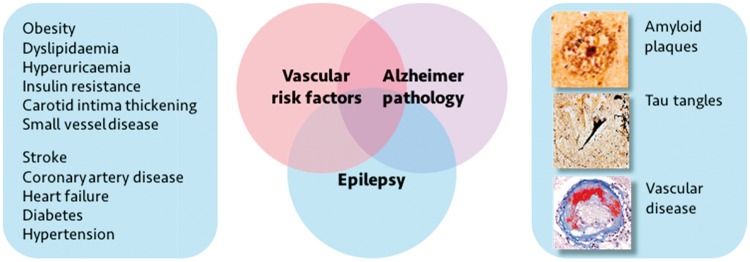
**The intersections of Alzheimer’s disease, epilepsy and vascular disease.** Several overlapping pathologies (*right*) can contribute to development of late-onset epilepsy as well as the development of dementia. In particular, vascular risk factors (*left*) are common in people with epilepsy. These may represent modifiable risk factors for both the development of dementia and of epileptogenesis.

A reason for postulating that this might indeed be the case is that it is increasingly becoming apparent that an important potential explanation for the co-occurrence of both seizures and dementia is that these conditions share common risk factors (Fig. 2). Several features have been associated with accelerated cognitive decline, brain ageing and dementia. These include increased vascular risk factors such as hypertension, diabetes, obesity, smoking and low exercise, all of which predispose to atherosclerosis; altered lifestyles such as decreased social interaction and physical inactivity; treatment with medications (including some anti-epileptic medications) that adversely affect cholesterol, folate and glucose metabolism; as well as elevated inflammatory markers (Schwaninger *et al.*, 2000; Fratiglioni *et al.*, 2004; Dik *et al.*, 2005; Vezzani and Granata, 2005; Panza *et al.*, 2006; Hamed, 2014).

While many of these are over-represented in patients with epilepsy (Hermann *et al.*, 2008; Hamed, 2014; Keezer *et al.*, 2016), the relationships of these factors to cognitive and brain ageing are only now beginning to be systematically examined. For example, while it has been long recognized that late onset epilepsy associates with an increased risk of stroke (Cleary *et al.*, 2004; Wannamaker *et al.*, 2015), it is only more recently that the extent of cerebrovascular disease has come under scrutiny in this group of patients (Gibson *et al.*, 2014; Hanby *et al.*, 2015).

An important contribution to this area was made by Sillanpää *et al.* (2015), who followed a cohort of 245 patients with childhood onset epilepsy living in the vicinity of the Turku University Hospital, Finland, for over 50 years. The authors demonstrate that even in ‘uncomplicated epilepsy’, namely patients without major neurological impairment at onset, MRI at 50-year follow-up demonstrated significantly more evidence of cerebrovascular disease than in controls. These findings could not be correlated to lifestyle choices such as smoking, alcohol consumption or physical exercise or to standard vascular risk factors such as hyperlipidaemia, diabetes or hypertension. Even patients with primary generalized epilepsy, most of whom were in terminal remission from seizures, still showed increased markers of cerebrovascular disease on brain imaging. One may speculate from these data that the increased vascular burden may be due to epilepsy itself, be that the underlying aetiology of the epilepsy, consequences of recurrent seizures (for example repeated head injury or bouts of status epilepticus), or chronic exposure to AEDs.

The same group has now also published data from amyloid PET imaging, showing that some of the Turku patients have positive evidence of amyloid deposition, with greater frequency than healthy controls, particularly in prefrontal cortex (Joutsa *et al.*, 2017). Higher amyloid deposition occurred in those individuals who were *APOE4* genotype positive—which also puts healthy individuals at greater risk of developing Alzheimer’s disease as well as vascular dementia (Liu *et al.*, 2013; Sun *et al.*, 2015). The impact of these imaging findings on cognition in this valuable cohort awaits further study.

As a corollary to such investigations on young-onset epilepsy cases who were imaged later in life, Maxwell *et al.* (2013) retrospectively examined 105 older patients with late onset epilepsy or isolated seizures and 105 controls. Radiological evidence of cerebrovascular disease, both large and/or small vessel disease, was more prevalent in people with seizures (Maxwell *et al.*, 2013). Notably, though, the majority of cases and controls had undergone CT brain imaging, potentially reducing the sensitivity of detecting small vessel disease (Maxwell *et al.*, 2013). The same group subsequently went on to examine MRI features of occult cerebrovascular disease in a small cohort of patients with late onset epilepsy and age-matched controls, reporting that the cases with epilepsy had lower cortical volume, especially in the temporal lobes, and an increased volume of white matter hyperintensity. There were additional changes in a measure of arterial arrival time, which led the authors to suggest that occult cerebrovascular disease might therefore contribute to late onset epilepsy (Hanby *et al.*, 2015).

Although this seems plausible, it remains unclear whether modification of vascular risk factors would reduce seizures (beyond the benefit of preventing clinically apparent strokes) and/or mitigate against cognitive decline in patients with epilepsy. The results of a recent study of Seventh Day Adventists and Baptists in Denmark—religious groups that desist completely or refrain from excessive consumption of alcohol and tobacco—demonstrated that there was a reduced incidence of Alzheimer’s disease in these communities, but without any change in the standardized incidence rate of epilepsy (Thygesen *et al.*, 2017). These findings suggest Alzheimer’s disease and seizure incidence can be dissociated, but what is required is a well-designed prospective clinical trial to establish whether tight control of vascular and lifestyle risk factors has an impact upon cognitive decline and incidence of dementia in people with epilepsy. It would also be helpful to disentangle how much of the cognitive deficit observed in an older individual with epilepsy relates to epilepsy (seizures and IEDs) and how much is attributable to underlying pathological changes, including vascular pathology (Hamed, 2014), thereby potentially enabling stratification and appropriate targeting of therapeutics.

## Molecular links between epilepsy and dementia in older patients

Another possible explanation for the increased risk of dementia in epilepsy is that some dementia syndromes might also share underlying pathological mechanisms with epilepsy, or that seizures might trigger pathological changes that make the brain more vulnerable to developing dementia pathology. Limited histopathological work has been performed to better delineate these aspects. In their study examining 138 people with chronic epilepsy, Thom *et al.* (2011) found that there was a higher incidence of cerebrovascular disease in older patients, and a significant correlation with cerebrovascular disease and higher Braak stages of Alzheimer’s disease-type pathology. However, when age was factored out, the association between cerebrovascular disease and Braak stage was no longer significant (Thom *et al.*, 2011). Importantly in this cohort of patients, 30% had evidence of traumatic brain injury and this, rather than the number of seizures, correlated with a higher Braak stage (Thom *et al.*, 2011). Similarly it has long been recognized that traumatic brain injury can increase the rate of dementia (Gualtieri and Cox, 1991). However, how traumatic brain injury, dementia and epilepsy, which may directly cause head injury through a seizure or indeed be a consequence of a previous head injury, intersect remains incompletely determined.

At a molecular level, deregulation of kinases, for example cyclin dependent kinase 5, which is known to be important in Alzheimer’s disease, has been shown in epileptogenic lesions (Sen *et al.*, 2006, 2007*b*). Furthermore, immunohistochemical analysis of tissue from some patients with epilepsy secondary to focal cortical dysplasia reveals aggregation of tau, similar to that in Alzheimer’s disease (Sen *et al.*, 2007*a*). Such changes in tau aggregation were observed only in the older individuals of this small sample, and were limited to the epileptogenic lesion alone, not being seen in adjacent histologically-normal cortex. In the two oldest patients in the series, 3-repeat (3R) and 4-repeat (4R) tau tangles were detected with a potential increase in the 4R:3R ratio. These two older individuals did also demonstrate amyloid-β positivity. Thus molecular cascades important in both seizure generation and neurodegeneration might converge within epileptogenic tissue in older patients (Sen *et al.*, 2008).

A recent report of patients aged over 50 years undergoing temporal lobectomy for hippocampal sclerosis (*n* = 33; age 50–65 years) documented that the majority demonstrated abnormal tau immunohistochemistry (Tai *et al.*, 2016). Of the 24 cases with AT8 (a monoclonal antibody that recognizes tau) positivity, 10 had a Braak-like pattern and eight had features more compatible with chronic traumatic encephalopathy. Several cases, though, had unusual patterns of AT8 immunopositivity with, for example, subpial band staining and unusual granulations. The CA1 sector of the hippocampus seemed less involved than in patients with Alzheimer’s disease, although it should be noted that hippocampal sclerosis, the pathology underlying the epilepsy in all resected cases, can result in severe neuronal loss through CA1. Both 3R and 4R isoforms of tau were present. Tau burden correlated with postoperative, but not preoperative, cognitive scores and although it is difficult to infer much from a single case, the patient with the highest burden of tau pathology in resected tissue went on to develop Alzheimer’s disease 9 years after epilepsy surgery.

There has perhaps been less attention given to amyloid-β deposition in patients with epilepsy overall. Increased levels of amyloid-β precursor protein (APP) (Sheng *et al.*, 1994) and age-accelerated presence of senile amyloid plaques (Mackenzie and Miller, 1994) have been observed in temporal lobectomy tissue from older TLE cases and seizures are particularly prevalent in patients with Alzheimer’s disease with *APP* duplication compared to other dominant Alzheimer’s disease genetic mutations (Zarea *et al.*, 2016). The expression of APP, but not the level of *APP* mRNA, is higher in temporal lobe and hippocampi from patients undergoing epilepsy surgery compared to controls (Sima *et al.*, 2014). Nonetheless, in human epileptogenic tissue, amyloid-β pathology is not often detected. Notably Tai *et al.* (2016) found that 28/33 of their relatively older surgical cases did not demonstrate any staining for amyloid-β and in three cases amyloid-β staining was sparse. These data are similar to the earlier study examining post-mortem samples from patients with chronic epilepsy (Thom *et al.*, 2011) where only 34% of 138 patients demonstrated amyloid-β positivity and only 8% of the whole cohort had frequent plaques. Taken together, data from human studies might suggest that chronic temporal lobe epilepsy can associate with a tauopathy and while tau burden may contribute to cognitive difficulties, the molecular mechanisms underlying epilepsy-associated tau pathology might differ from those that drive Alzheimer’s disease.

Animal models of Alzheimer’s disease are also providing possible insights into commonalities of network disruption in Alzheimer’s disease and epilepsy (Scharfman, 2012). Fibrillar amyloid-β can disrupt neuronal membrane properties and trigger epileptiform activity (Westmark *et al.*, 2008; Minkeviciene *et al.*, 2009). Furthermore, in double-transgenic APP23xPS45 mice [which overexpress both APP (APPSwe) and mutant presenilin-1] there is a significant increase in hyperactive neurons, specifically adjacent to amyloid plaques (Busche *et al.*, 2008). This appears to be associated with disruption of the balance of excitation and inhibition at long range, across brain networks (Busche and Konnerth, 2016; Palop and Mucke, 2016). Remarkably, levetiracetam and topiramate, two widely prescribed AEDs, reduced amyloid plaques in this model (Shi *et al.*, 2013). In addition, both AEDs improved spatial memory in the transgenic mice tested on the Morris water maze (Shi *et al.*, 2013).

**Figure 3:**
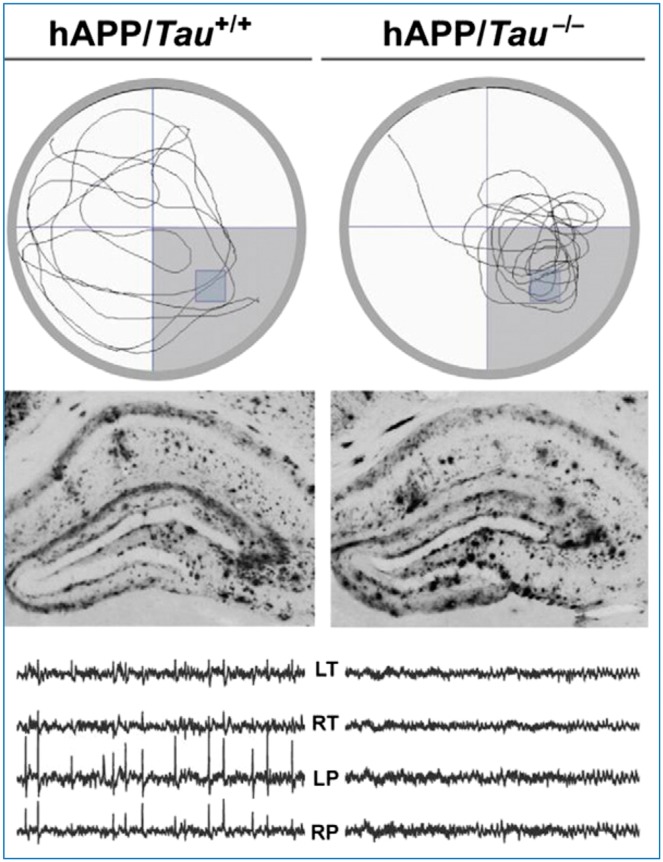
**Mouse model of Alzheimer’s disease shows abnormal spiking.** The human amyloid precursor (hAPP) transgenic mouse of model of Alzheimer’s disease shows deficits of spatial memory on the Morris water maze task, taking longer to find the platform (*top left*) and with abnormal spiking activity over left and right temporal and parietal cortex (*bottom left*). Reduction of tau by creating a hAPP mouse, which does not produce tau (hAPP/*Tau*^-/-^) normalizes both spatial memory and the EEG (*top* and *bottom right*, respectively), despite the fact that both models have similar amyloid plaque deposition (*middle*). Modified with permission from Roberson *et al.* (2011).

Other recent studies using a different mouse genetic model of Alzheimer’s disease [human APP (hAPP) transgenic] reported that these animals develop abnormal electrical activity in the hippocampus (Sanchez *et al.*, 2012). Further investigations on this animal model have revealed that deficits in spatial memory and abnormal spiking on EEG can be normalized when the mouse is also depleted of tau (by knockout of the tau gene, *MAPT*), without any effect on amyloid deposition (Roberson *et al.*, 2007) (Fig. 3).

Treatment with levetiracetam—but not other tested AEDs (ethosuximide, gabapentin, phenytoin, pregabalin, valproic acid and vigabatrin)—reduced such aberrant electrical activity, and chronic treatment with levetiracetam actually improved memory performance and behaviour in these mice (Sanchez *et al.*, 2012). Electrophysiological studies in acute hippocampal slices confirmed that levetiracetam reversed deficits in synaptic transmission restoring long-term potentiation curves so that they were no different to that from non-transgenic mice (Sanchez *et al.*, 2012). Importantly, higher doses of levetiracetam were ineffective and once the drug was withdrawn there was recurrence of epileptiform activity with associated return of the behavioural and cognitive deficits. In humans, levetiracetam has also been shown to suppress aberrant hippocampal blood oxygen level-dependent (BOLD) hyperactivity in amnestic mild cognitive impairment individuals, with associated improvement on an experimental memory task (Bakker *et al.*, 2012), as discussed in more detail below in the ‘Network level effects in epilepsy and dementia’ section.

As an elegant corollary of this work, several groups have investigated whether disruption of tau might have an impact in animal models of genetic epilepsy. For example, Dravet syndrome is a devastating epileptic encephalopathy that associates with mutations in the human *SCN1A* gene encoding the voltage gated potassium channel subunit Na_v_ 1.1. In the mouse model of Dravet syndrome, deletion of tau alleles decreased the number of seizures and reduced mortality (Gheyara *et al.*, 2014). There was also evidence of improved learning and memory with tau depletion (Gheyara *et al.*, 2014). In *Kcna*^−/−^ mice, which lack K_v_1.1 rectifier currents and whose neurons are therefore hyperexcitable, depletion of tau reduced seizure frequency and duration. While similarly in acquired models of epilepsy, depletion of tau through antisense oligonucleotides reduced the effect of chemoconvulsants (DeVos *et al.*, 2013) and constitutional depletion of tau through genetic deletion prevented deficits in spatial memory and learning after repeated mild head injuries (Cheng *et al.*, 2014). Finally, treatment with sodium selenate, a drug that reduces tau hyperphosphorylation, decreased seizure severity/frequency in three separate rodent models of epilepsy (Jones *et al.*, 2012). Taken together this body of work again illustrates commonality of pathways that may underpin epilepsy and dementia with demonstration that suppression of tau hyperphosphorylation or reduction in seizures of can benefit epilepsy and Alzheimer’s disease, respectively.

## Network level effects in epilepsy and dementia

Increasingly, epilepsy is considered to be a brain network disorder, with widespread functional connectivity changes associated with even focal pathologies (Dinkelacker *et al.*, 2016; Englot *et al.*, 2016). Moreover, it is now also appreciated that a major contribution to cognitive impairment in people with epilepsy is seizure activity, regardless of the underlying pathological cause of seizures (Holmes, 2015). In fact, although it has long been acknowledged that status epilepticus or recurrent seizures can impact upon cognition, findings in both animal models and humans demonstrate that interictal spikes or IEDs can also disrupt cognitive function (Kleen *et al.*, 2010, 2013) even if remote to the ictal onset zone (Ung *et al.*, 2017).

**Figure 4:**
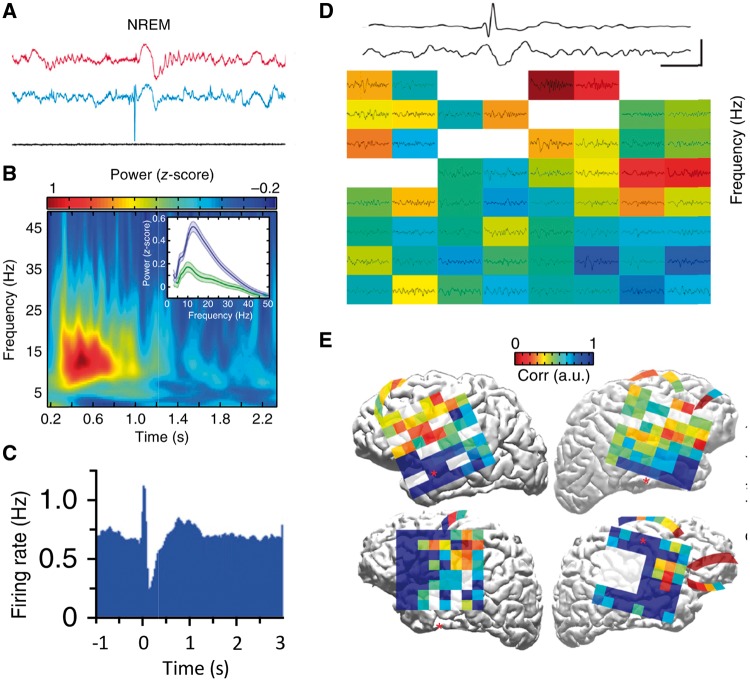
**Hippocampal interictal epileptiform discharges couple with frontal activity.** Rodent studies: (**A**) Medial prefrontal cortical (mPFC; red) and hippocampal (blue) local field potentials (LFPs) recorded during non-REM sleep in a kindling model of TLE. This is an example of a putative hippocampal IED-evoked spindle (oscillation) in frontal cortex. Similar findings occurred in REM sleep and awake states. (**B**) Normalized spectrogram in rat mPFC after hippocampal IED onset. The *inset* shows the change in averaged mPFC power spectrum from before (green) to after (blue) IED. (**C**) Average peri-event firing-rate histograms of mPFC pyramidal cells in the time window around hippocampal IED (which occurred at time zero) reveals a decrease in activity (‘down time’) in mPFC firing after the IED. Human studies: (**D**) LFP recorded from IED electrode in parahippocampal gyrus (*upper*) and subdural cortical electrode (*lower*) demonstrating time-locked spindle. The grid in frontal cortex shows *z*-scored spindle-band power across cortical electrocorticography (ECoG) array triggered on IED (white channels are non-functional). (**E**) ECoG grid and strip placement (each square represents one recording electrode) on the projected pial surface of four patients with epilepsy. Warm colours indicate high IED-spindle correlation; cool colours represent low correlation. Modified with permission from Gelinas *et al.* (2016).

In patients, the best evidence for this comes from investigation of individuals who have had depth electrodes implanted into their hippocampi for presurgical evaluation. IEDs were associated with significant impairment in recall on a working memory task (Kleen *et al.*, 2013; Ung *et al.*, 2017). A recent study in a rat model of TLE revealed hippocampal-medial prefrontal coupling changes associated with IEDs (Fig. 4A–C) (Gelinas *et al.*, 2016). Crucially, the frequency of IEDs and level of coupling correlated with memory impairment. Furthermore, a pilot study in patients with TLE demonstrated similar altered coupling between hippocampal IEDs and distant cortical electrical events, with IEDs leading to disruption of ongoing cortical activity (Fig. 4D and E) (Gelinas *et al.*, 2016). An independent, detailed examination of a case with idiopathic generalized epilepsy using simultaneous video-EEG and functional MRI while the patient performed a working memory task also revealed widespread cortical effects of IEDs (Chaudhary *et al.*, 2013), while a more recent group study on 67 cases reported that even IEDs originating outside the seizure zone can have a detrimental impact on memory (Ung *et al.*, 2017). Such findings support the view that IEDs can have long-range effects leading to disrupted cognitive function.

**Figure 5:**
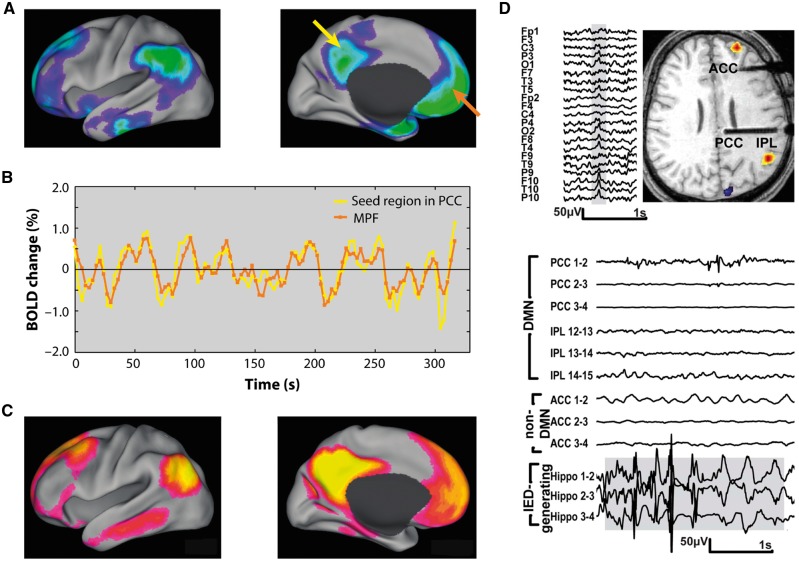
**Default mode network and epilepsy.** (**A**) DMN regions show decreases in activity when subjects perform cognitive tasks performance. (**B**) BOLD resting state activity is strongly correlated within DMN regions. Here activity is shown for the seed region in posterior cingulate cortex (yellow arrow in **A**) and another region which shows a similar pattern of activity, in medial prefrontal cortex (orange arrow in **A**). (**C**) Functional connectivity across DMN regions defined by spatial coherence in resting state BOLD signal fluctuations across these areas. (**D**) *Top* panel shows scalp EEG with right temporal IED and associated BOLD changes in a patient with TLE. Functional MRI demonstrates significant simultaneous activations in right frontal and parietal lobes and deactivation in cuneus. *Bottom* panel shows stereotaxic intracerebral EEG (SEEG) traces illustrating runs of hippocampal IEDs (*lower* three traces) and SEEG in posterior cingulate cortex (PCC), inferior parietal lobule (IPL) and anterior cingulate cortex (ACC). The most medial channel in PCC (*upper* trace) shows propagation of epileptic activity. (**A**–**C**) Adapted with permission from Raichle (2015); (**D**) Adapted from Fahoum *et al.* (2013).

One specific question about the nature of long-range network effects of IEDs and seizure activity is whether these might have effects via certain critical nodes in the brain. In particular, a great deal of attention has turned to the role of the so-called default mode network (DMN; Fig. 5) (Raichle, 2015). This ensemble of brain regions includes the posterior cingulate cortex, precuneus, lateral parietal and medial frontal regions, and is strongly linked to the hippocampus (Vincent *et al.*, 2006). The DMN is most active at rest or when an individual is not performing a specific experimenter-defined task, but crucially deactivates during goal-directed behaviour (Fig. 5A), as assessed for example by changes in the BOLD signal detected by functional MRI. Greater deactivation of the DMN correlates positively with better performance on cognitive tasks, but is negatively correlated with activation in brain regions such as dorsolateral prefrontal regions, which are considered to essential to performing the cognitive task (Sambataro *et al.*, 2010; Dang *et al.*, 2013). In addition, stronger functional connectivity between DMN brain regions (Fig. 5B and C) is also related to better scores on tests of episodic memory and executive function (Sambataro *et al.*, 2010; He *et al.*, 2012; Dang *et al.*, 2013).

In TLE, simultaneous functional MRI-EEG studies reveal that the medial temporal lobe BOLD signal is increased in association with IEDS (Laufs *et al.*, 2007; Zhang *et al.*, 2010). Furthermore, both structural connectivity (measured by diffusion weighted MRI) and functional connectivity (indexed by functional MRI) are decreased within the DMN, compared to healthy individuals (Liao *et al.*, 2011). Recent investigations of epilepsy patients using either functional MRI and/or EEG provide further support for abnormal DMN connectivity (Fig. 5D), either in the presence (Fahoum *et al.*, 2013) or absence of IEDs (Coito *et al.*, 2016; Robinson *et al.*, 2017). Thus there is now abundant evidence for long-distance effects on the DMN in people with epilepsy including TLE, both during IEDs and in their absence.

Intriguingly, similar patterns of findings implicating the DMN have emerged independently in the Alzheimer’s disease literature. Hyperactivity within the hippocampus when people perform memory tasks in association with decreased resting functional connectivity of the DMN has been reported in mild cognitive impairment (Dickerson *et al.*, 2005; Celone *et al.*, 2006; Bakker *et al.*, 2012), i.e. individuals who are at high risk of developing Alzheimer’s disease. Moreover, the level of DMN deactivation correlated with recall performance (Celone *et al.*, 2006). The presence of concomitant cerebrovascular disease in Alzheimer’s disease may lead to systematic network changes that extend beyond the DMN, involving brain regions implicated in executive functions (Chong *et al.*, 2017).

It is now also well established that amyloid deposition in Alzheimer’s disease (as measured using amyloid PET imaging) follows a pattern of distribution in the brain that is strikingly similar to the DMN. Remarkably, even in healthy older individuals, both hippocampal hyperactivity and reduced DMN deactivation during memory encoding tasks are associated with greater amyloid deposition (Sperling *et al.*, 2009; Mormino *et al.*, 2012). More recent investigation using tau PET imaging also reveals hyperactivity within the medial temporal lobe associated with greater tau deposition (Marks *et al.*, 2017). Further, a longitudinal study using both functional MRI and amyloid PET imaging has reported that greater hippocampal activation on a memory task at baseline was associated with increased amyloid deposition over time (Leal *et al.*, 2017).

It has been difficult to determine if hippocampal hyperactivity is pathological or simply an indicator of a compensatory response for declining memory in patients with mild cognitive impairment or those with greater amyloid/tau deposition. A proof-of-concept study demonstrated that 2 weeks of levetiracetam (at 125 mg twice a day or 62.5 mg twice a day but not at higher doses of 250 mg twice a day) suppressed aberrant hippocampal BOLD hyperactivity in amnestic mild cognitive impairment individuals, associated with a significant improvement in memory performance on an experimental task (Bakker *et al.*, 2012). These findings therefore raise the possibility that hippocampal hyperactivity represents abnormal, pathological activity rather than a compensatory mechanism, as also suggested by findings in animal models of Alzheimer’s disease (Palop and Mucke, 2016). In the TLE literature, an independent study reported that levetiracetam normalized failure of hippocampal deactivation during a working memory task (Wandschneider *et al.*, 2014), again pointing to important parallels between Alzheimer’s disease and TLE.

In more severe mild cognitive impairment and Alzheimer’s disease cases hippocampal activity actually falls below normal, but DMN functional connectivity remains weakened (Celone *et al.*, 2006). In addition, established Alzheimer’s disease not only has an increased associated risk of developing epilepsy (Imfeld *et al.*, 2013), but recent work has revealed that patients with Alzheimer’s disease also show significantly greater subclinical epileptiform activity. One investigation has documented silent hippocampal seizure activity recorded from foramen ovale intracranial electrodes in patients with Alzheimer’s disease (Lam *et al.*, 2017). In a different study, ∼42% of Alzheimer’s disease cases monitored using overnight video-EEG had evidence of epileptiform activity (Vossel *et al.*, 2016). This was defined as paroxysmal sharp waveforms of 20–200 ms, distinct from ongoing background activity and associated with a subsequent slow wave. Interestingly, there was no significant difference in brain atrophy between Alzheimer’s disease cases with and without subclinical epileptiform activity, suggesting that severity of Alzheimer’s disease might not be the crucial factor in distinguishing between these groups. Therefore, several lines of evidence point to intriguing and increasing similarities in network dysfunction associated with epilepsy—particularly TLE—and Alzheimer’s disease, raising the possibility that stabilization of abnormal neuronal networks might not only reduce seizures, but also improve cognition.

## Conclusions and future directions

In this review we have sought to better understand whether seizures promote cognitive impairment and/or dementia, whether dementia causes seizures, or if common underlying pathophysiological mechanisms are responsible for both? The findings to date show that older adults with epilepsy, whether they developed the condition at a younger age or *de novo* later in life, exhibit poorer performance across a range of cognitive measures compared to healthy controls (Table 1) and have an increased risk of developing dementia. However, we note with caution that there has been surprisingly little investigation specifically of cognitive function in late-onset cases. The data that are available show that there is clear heterogeneity in cognitive performance across this group (Witt *et al.*, 2014), just as there is in younger people with one subtype of epilepsy, TLE (Hermann *et al.*, 2007). The key factors that determine this variation remain to be established and would be an important issue for future studies to focus on. Stratification of dementia risk in people with epilepsy at an early stage would potentially have a major impact in this field.

Although some pathologies (e.g. cerebrovascular disease) appear to have a worse prognosis for cognition, how different underlying pathologies interact with cognitive reserve, seizure control, psychological co-morbidities and lifestyle or vascular risk factors to impact upon everyday life, for both patients and carers, remains to be definitively established. Whether the pattern of cognitive impairment can help to stratify dementia risk or identify patients with more vascular versus Alzheimer-like pathology has also not been definitively assessed. However, the likelihood is that with mixed pathologies (e.g. traumatic, vascular, tau, amyloid) there might not be pure phenotypes. Indeed it is unclear whether dementia in the context of epilepsy represents a very different condition to dementia in other disorders, such as Alzheimer’s disease, vascular dementia and mixed Alzheimer’s disease/vascular dementia, or whether specialized criteria for mild cognitive impairment or dementia diagnosis in people with epilepsy might need to be introduced in the future.

Whether epilepsy simply lowers brain reserve and thereby facilitates manifestation of dementia-related pathology, or whether epilepsy is a disorder that itself produces dementia—by the effects of seizures and IEDs on brain structure and function—also remains unclear. The evidence reviewed here certainly suggests that some vascular risk factors and pathophysiological mechanisms (e.g. tau and vascular pathology) are indeed common to people with epilepsy and dementia.

Another important future direction of research would be to investigate whether more aggressive management of vascular risk factors (e.g. blood pressure, smoking, diet and exercise) would improve control of seizures and thereby protect against worsening of cognitive decline in people with epilepsy. Likewise, an important related, unanswered question is whether elderly patients with epilepsy are genuinely susceptible to accelerated cognitive decline (Fig. 1). Does the pathological burden (vascular, traumatic, tau or amyloid) in people with epilepsy interact synergistically with ongoing seizure activity or IEDs to lead to progressive worsening of cognitive function and further deviation away from the normal trajectory associated with cognitive ageing? Longitudinal studies of older patients with epilepsy could potentially assist greatly in this regard.

Whether dementia causes seizures has also become a topic of great interest recently. Again, as reviewed here, there is increasing evidence from both human and animal model studies of an association between tau and/or amyloid pathology and development of seizures. In addition, some AEDs appear to have a beneficial impact not only in reducing such activity, but in actually improving cognitive function, perhaps by long-distance effects at the level of brain networks. Whether AED therapy would have a beneficial effect in patients with Alzheimer’s disease or mild cognitive impairment who do not have overt seizures is the subject of ongoing trials.

All these questions are unlikely to be answered by small-scale studies. Given the range of underlying pathologies and the multitude of factors that impact upon cognitive performance discussed here, there is a pressing need for large-scale patient registries with harmonized systems of cognitive assessment. Standardization across centres as well as across disease diagnoses would greatly facilitate such an endeavour. Development of short, pragmatic test batteries that are not culturally specific would also improve detection of cognitive impairment in elderly people with epilepsy in the developing world. Baseline and longitudinal tracking of cognitive function using standardized neuropsychological measures is a particularly important consideration in this population in whom, as illustrated, very little research has been conducted to date.

In this review we have tried to identify what is currently understood about cognitive deficits in older people with epilepsy. We have highlighted the commonality of molecular and brain network disruption that may bind epilepsy and dementia much more closely than previously thought. We have also sought to identify possible areas of future research that may, in due course, offer the opportunities of slowing cognitive decline in patients with epilepsy.

## Abbreviations

AED: anti-epileptic drug
DMN: default mode network
IED: interictal epileptiform discharge
TLE: temporal lobe epilepsy
